# Managing macromolecular crystallographic data with a laboratory information management system

**DOI:** 10.1107/S2059798324005680

**Published:** 2024-07-10

**Authors:** Edward Daniel, Rik K. Wierenga, Lari Lehtiö

**Affiliations:** a Biocenter Oulu and Faculty of Biochemistry and Molecular Medicine University of Oulu Oulu Finland; Global Phasing Ltd, United Kingdom

**Keywords:** data management, crystallography, laboratory information management systems, LIMS, *IceBear*, FAIR data

## Abstract

Protein crystallography is an established method to study the atomic structures of macromolecules and their complexes. A prerequisite for successful structure determination is diffraction-quality crystals, which may require extensive optimization of both the protein and the conditions, and hence projects can stretch over an extended period, with multiple users being involved. The workflow from crystallization and crystal treatment to deposition and publication is well defined, and therefore an electronic laboratory information management system (LIMS) is well suited to management of the data. Completion of the project requires key information on all the steps being available and this information should also be made available according to the FAIR principles. As crystallized samples are typically shipped between facilities, a key feature to be captured in the LIMS is the exchange of metadata between the crystallization facility of the home laboratory and, for example, synchrotron facilities. On completion, structures are deposited in the Protein Data Bank (PDB) and the LIMS can include the PDB code in its database, completing the chain of custody from crystallization to structure deposition and publication. A LIMS designed for macromolecular crystallography, *IceBear*, is available as a standalone installation and as a hosted service, and the implementation of key features for the capture of metadata in *IceBear* is discussed as an example.

## Abstract 2

It is discussed how the metadata for the workflow from protein crystallization to the deposition of crystal structures can best be captured by using a dedicated but flexible laboratory information management system that can be either a standalone installation or a hosted service to mitigate the IT risks.

## 1. Introduction

**Figure 1:**
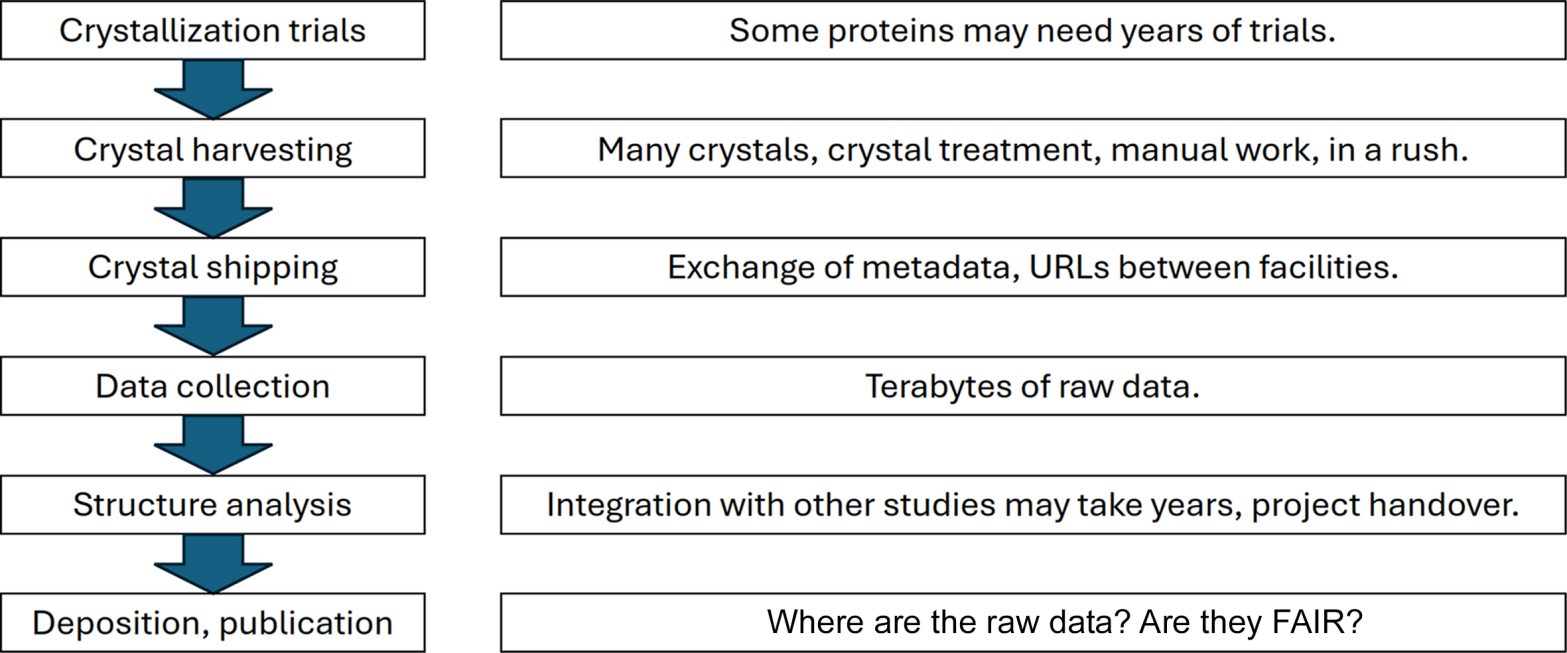
Typical crystallography workflow and key data-management time points for data collection from crystals grown in a home laboratory and subsequently used for data collection at a synchrotron. This protocol starts from recording the sequence of the construct used and the crystallization conditions and is completed by deposition of the structure, the structure factors and the raw images. The very common workflow includes the exchange of samples and metadata between the crystallization facility at the home laboratory and the data-collection beamline at the synchrotron, as captured by the ‘Crystal shipping’ box.

Protein crystallography has impacted our understanding of biological processes, making it possible to carry out structure-based drug discovery and enabling the development of artificial intelligence methods to predict protein structures with high precision (Jumper *et al.*, 2021 ▸). The latter is a result of decades-long tradition and the requirement for researchers to deposit processed data, *i.e.* structure factors and atomic coordinates, in the Protein Data Bank for open access (Berman *et al.*, 2000 ▸). While protein crystallography itself is a mature process and in some cases can be conducted in an automated fashion with high throughput, there remains an element of uncertainty in obtaining crystals in the first place. While some proteins may crystallize immediately when mixed with appropriate precipitants, particularly difficult proteins may require years of trials and optimization of both the sample and crystallization conditions (Fig. 1 ▸).

Due to ongoing advances at the X-ray beamlines at synchrotrons, particularly in the areas of detector resolution and sample throughput, the amount of data generated in a typical synchrotron session has been increasing over time (Lynch *et al.*, 2023 ▸). Currently, multiple terabytes of data can be generated in a single session of typically 8 h. At the European synchrotrons the data are managed by *ISPyB* (Delagenière *et al.*, 2011 ▸). Although automated structure-solution pipelines may solve the structure in minutes, it is also possible for several years to elapse between data collection and structure solution and final refinement (Fig. 1 ▸). The metadata and the raw data must be kept safe and organized throughout the process (Helliwell, 2022 ▸; Kroon-Batenburg *et al.*, 2024 ▸; Haquin *et al.*, 2008 ▸). Synchrotrons may store the data and even make them open access after a certain embargo period, but data policies differ between facilities. The publication process may require the use of a number of additional methods and integration of the results from, for example, cell studies with the information on the solved structure, with the consequence that the time from data collection to publication may be years, even if the structure itself is solved quickly. As an example, it is therefore entirely possible that the synchrotron beamline may have upgraded its detector in the meantime, meaning that the equipment at the time of deposition may not be that on which the data set was collected. The recording of such information at the time of data collection is therefore essential.

The rise of FAIR data (FAIR: Findable, Accessible, Interoperable, Reusable) poses further challenges for tracking the experiments, as a PDB deposition of coordinates and structure factors may no longer be sufficient (Wilkinson *et al.*, 2016 ▸). The entire history of crystallization and data collection must be trackable and ideally made available in a format that allows it to be freely understood and reproduced. As this work may involve terabytes of data generated over several years, as well as several researchers, it is important to organize these data from the outset. A transition from paper notebooks to digital systems facilitates the availability of the experimental notes and data remarkably, and in general there are two main options for keeping track of these data: laboratory information management systems (LIMS) and electronic laboratory notebooks (ELNs). Each has its own advantages and dis­advantages.

An ELN is typically more lightweight than a LIMS, with significantly more flexibility in how experimental data can be recorded. This makes it well suited to highly fluid processes with constantly changing requirements for bookkeeping. However, while this flexibility can follow the experimental workflow of an individual scientist, it can make it more difficult to mine the data later. By contrast, a LIMS is typically highly structured and tailored to a well defined experimental flow. It takes advantage of the stability of workflows by offering a user interface that is highly optimized for the specific tasks being performed, as well as by integrating tightly with laboratory equipment. Its underlying database structure is likely to be as task-optimized as its user interface, sacrificing flexibility for minability.

This distinction may not always be clear; a well featured ELN may well encroach upon the territory of LIMS, while some LIMS may offer a degree of flexibility approaching that of an ELN. However, given that traditional macromolecular crystallography techniques are mature enough to have been developed into high-throughput and highly automated processes (Fig. 1 ▸), the tight coupling of the LIMS to the experimental workflow is more of an advantage than the flexibility of the ELN, and we will therefore address recent developments of LIMS throughout this brief overview. Examples of LIMS software used for structural biology are listed in Table 1 ▸. In particular, we will refer to the *IceBear* LIMS (https://www.icebear.fi/) and its recent updates developed by the authors (Daniel *et al.*, 2021 ▸).

## 2. Results and discussion

### 2.1. Managing proteins and projects in a LIMS

A basic requirement of a protein crystallography LIMS is that it should record the protein sequence being worked on, along with the various constructs used to express the typically recombinant proteins and protein fragments. The LIMS should also handle assemblies consisting of multiple proteins of a hetero-oligomer. To enable the tracking of metadata for ligand-binding studies, for example for studies of enzyme catalysis and drug discovery, the LIMS should have the possibility of tracking the ligands used for co-crystallization and soaking, at least at the level of notes. All of the subsequent steps of the workflow will be tied back to this basic information on the experimental setup. It is likely that some access restrictions to the information will be needed beyond a simple login, both to prevent unauthorized access to confidential work and to eliminate clutter.

In *IceBear*, these requirements are met by organizing the work into ‘projects’; we recommend, but do not require, that each protein be placed into its own dedicated project. Each protein has an acronym and one or more sequences provided by the project owner. Users are organized into groups, which are then granted read-only or full access to a project. Project handover from a leaving researcher to a new project member is easily achieved by assigning the new researcher the access rights to the appropriate project.

### 2.2. Keeping track of crystallization trials

While the LIMS has a record of the amino-acid sequence of the protein constructs, it also needs information on the protein concentration, protein buffer (composition, pH) and volumes used in the crystallization experiments. Similarly, a record should be kept of the precipitant solutions, including the concentrations, additives, pH and volumes used in the experiments to allow downstream optimization as well as reporting. Standard screens can be imported with a couple of clicks, but *IceBear* can import both optimization and standard screens in various formats (Daniel *et al.*, 2021 ▸). Recording this information is essential and researchers are reminded by *IceBear* in cases where it has not been provided.

In commercial and in-house crystallization screens the chemicals are currently not listed in a standardized format and this may limit some possible analysis later, which could become a future development target. A ligand may have been introduced to the protein prior to the experiment or added to the drops later, and this crystal-treatment information may be provided by the scientist. *IceBear* provides the flexibility to also handle this information as notes, allowing the users to define a chemical, for example an in-house inhibitor, by an identification code. *IceBear* includes information on crystallization plate types and knows the incubation temperature of the imager.

Monitoring of the crystallization drops is performed by visual inspection for days, weeks or even months. Automatic imaging of crystallization plates naturally makes this task easier for the scientists who inspect the drops, and who can also score the drops into different categories to keep track of the most promising conditions, and in the ideal case indicates in the LIMS the drops in which crystals are present. Scoring at least the best images in each inspection is recommended; not only does this highlight the wells with crystals in the plate overview, but also allows plates to be sorted by best score.

*IceBear* connects to the image stores and databases of automated imagers to import inspections as they occur. For smaller laboratories without imaging hardware, it is still important to preserve and organize microscope images. *IceBear* can work entirely without images if need be, but also allows the user to attach manually captured images obtained from a microscope. The times of the first and subsequent imaging events are recorded, allowing users to view time-lapsed, low-frame-rate movies of crystal growth using either visible or UV images.

Automated drop scoring by various artificial intelligence (AI) methods can also be integrated into the LIMS (Bruno *et al.*, 2018 ▸; Milne *et al.*, 2023 ▸) and this feature is currently under development for *IceBear*. Some imaging systems (Formulatrix/*Rock Maker*) offer this within their own ecosystem, while other AI scoring may be integrated into the LIMS with varying degrees of effort. Provided that the AI is trustworthy, such integration can greatly reduce the user workload by prioritizing interesting images of crystallization drops.

### 2.3. Crystal harvesting

Once crystals have grown, they are harvested either (i) directly via a cryocooling protocol and subsequent mounting on a pin or (ii) first used for a ligand-soaking experiment, followed by cryocooling and mounting on a pin. At this stage a unique sample name for each crystal needs to be generated. In most laboratories, crystal cryoprotection and mounting on a pin is still an entirely manual process. Cryocooling of the fished crystals is often carried out on a cramped bench near liquid nitrogen. Therefore, any LIMS work, such as specifying the drop from which the crystal is harvested and the pin barcode and/or its position in the puck and in the dewar, needs to be minimized while preserving the chain of custody. *IceBear* has a minimal but highly functional crystal-fishing interface that is optimized for use with a 2D barcode scanner to enter the bar codes of the pins, pucks and dewars into a database and drag–drop operations on a touch screen. Researchers can add detailed notes at this point, but to facilitate the process commonly used remarks can also be added with a single touch. The simplicity of the default workflow is intended to drive user compliance, ensuring that crystal harvesting is recorded correctly and enabling the subsequent submission of accurate shipment information.

Devices for automated crystal harvesting by cryocooling are available, for example the CrystalDirect (Cipriani *et al.*, 2012 ▸). Integration with *CRIMS* (Table 1 ▸) allows a crystal-mounting robot to harvest crystals from positions marked on drop images by the user, automatically recording the sample position within the puck. Such devices are well suited to high-throughput facilities. The Crystal Shifter from Oxford Lab Technologies is an elegant tool that, when properly integrated into a LIMS, ensures that manually fished crystals are assigned to the correct drop and its crystallization and soaking metadata (Wright *et al.*, 2021 ▸).

*In situ* data collection eliminates any need for crystal harvesting, with only the positions of the crystals needing to be transferred to the beamline. Indeed, fully integrated beamlines such as VMXi at Diamond Light Source can move plates between the imaging robot and the X-ray beam without any manual intervention, and data collection is guided based solely on the crystals marked for collection in the LIMS by the user (Thompson *et al.*, 2024 ▸).

### 2.4. Sending a shipment to the synchrotron

While the line between crystal harvesting and shipment assembly is somewhat blurred, at some point a dewar full of mounted crystals will be sent to the synchrotron. During the workflow, the fished crystals may first be used for soaking experiments related to ligand-binding studies before flash-cooling, may be put into storage dewar or may be placed directly into a shipping dewar. In parallel with the physical shipment, a virtual shipment-submission process must take place, informing the synchrotron *ISPyB* of the samples included, container barcodes *etc*. This is a tedious manual data-entry process, either directly into the synchrotron systems or by uploading a .csv file assembled in spreadsheet software. Most, if not all, of the information needed by the synchrotron will already be stored in the LIMS. Therefore, provided that the synchrotron provides a mechanism for doing so, the LIMS can assemble and submit the shipment information with minimal user interaction. *IceBear* supports shipment submission of metadata for synchrotrons running *ISPyB* (Oscarsson *et al.*, 2019 ▸; Fisher *et al.*, 2015 ▸). In addition, it can exchange its own URLs and database IDs with the synchrotron, allowing seamless navigation between home-laboratory and synchrotron records of the sample as well as informing on the location of the experimental data for later retrieval.

### 2.5. Data collection, data processing and structure solution at the synchrotron

**Figure 2:**
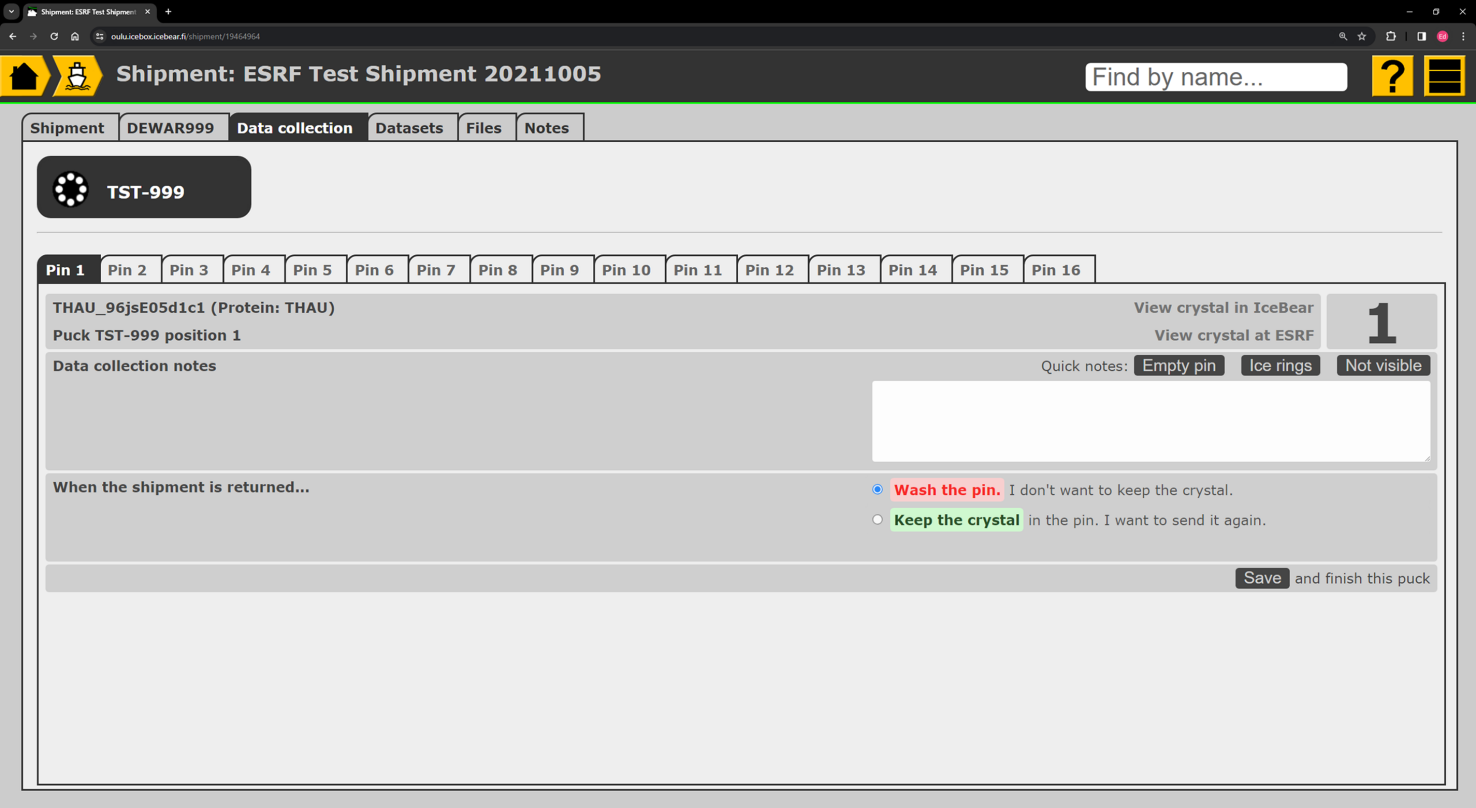
Making notes when collecting data. Notes can be captured immediately and are associated with the crystal. ‘Quick notes’ buttons allow commonly used notes to be added easily.

**Figure 3:**
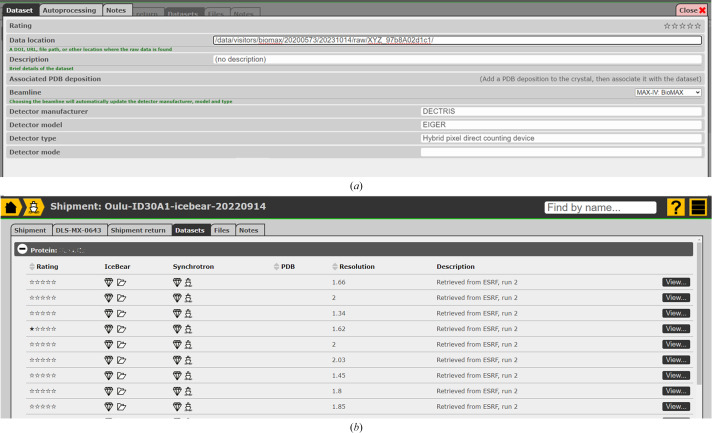
Retrieval of metadata as a new feature in *IceBear*. (*a*) Details of the experimental setup for data collection, including the location of the raw data and details of the beamline used. (*b*) List of data sets obtained from crystals of a shipment, sorted from high to low resolution. For each data set the icons provide the link to its crystal page and its project page (in the *IceBear* database), as well as to its *ISPyB* crystal page and its *ISPyB* shipment page.

In the case of manual data collection at the synchrotron beamline, *IceBear* allows notes to be made on each crystal that is being used for data collection (Fig. 2 ▸). Most synchrotrons have data-processing pipelines, and information on the results of this process is typically stored in the *ISPyB* database, which is accessible through the web browser. Downstream processing pipelines may also provide automatic structure solution and evaluation of possible bound ligands in the crystal structure if information concerning the relevant sequences, structures and ligands is available. The metadata from the results of the data collection provided by the data-processing pipelines, along with the detector details and the location of the raw data, can be retrieved from *ISPyB* by the home-laboratory LIMS, associating the data-collection results to the particular harvested crystal. This feature is currently being implemented in *IceBear* and is already functional for some information for some synchrotron facilities (Fig. 3 ▸), requiring only that the user provide their *ISPyB* credentials.

### 2.6. Deposition of the crystal structure

**Figure 4:**
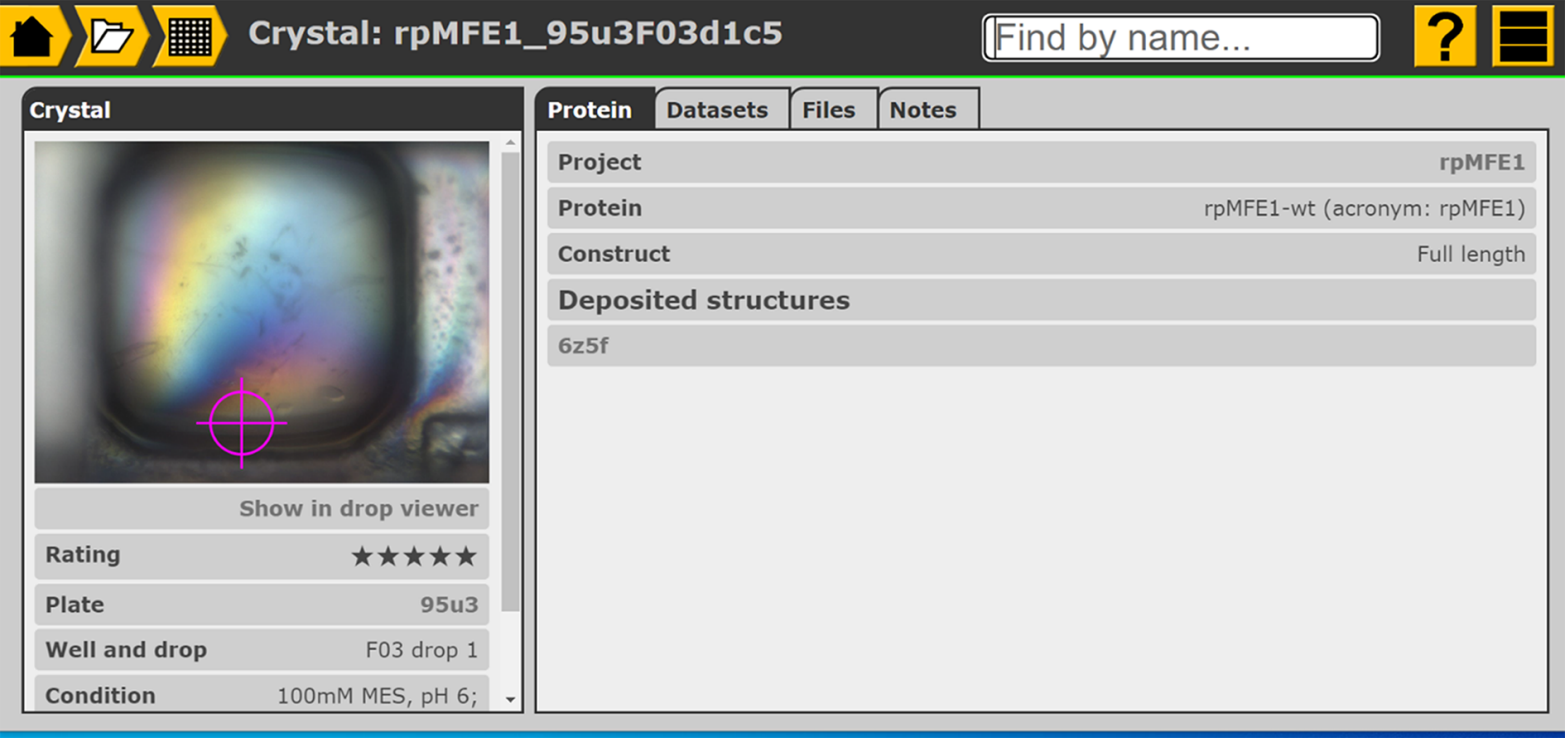
The crystal page of *IceBear* is its central hub, with pointers to the construct used, the crystallization and crystal-treatment information, the PDB and the location of the raw data set. The files option allows, for example, the archiving of important log files relevant to this crystal. The notes are generated either by *IceBear* (for example when a crystal is harvested and when a crystal is shipped) or by the user (at any point). The crystal page of a crystal can be found by using the search option (top right), for example by specifying its PDB code (6z5f; Sridhar *et al.*, 2020 ▸) or its sample name. The top left icons are navigation tools to find the plate and project information. The question mark (top right) provides context-specific help information.

Recording the structure deposition in the LIMS by providing the PDB code on the crystal page (Fig. 4 ▸) in *IceBear* associates the PDB code with the relevant data sets and crystals, and completes the chain of custody from protein sequence and crystallization conditions to deposited structure. All information relevant to this structure can be found with easy-to-use navigation tools (Fig. 4 ▸). On the PDB page links are provided to the EBI, RCSB, PDBj and Proteopedia (Hodis *et al.*, 2008 ▸). The deposition process currently requires manual entry by the researchers, while *IceBear* provides key information on the sample that can be added to the PDB records upon deposition. Being able to import data-collection information to *IceBear* will provide the depositor with easy access to, for example, the data-collection date and beamline and detector information.

The raw data are critical information that must be preserved. *IceBear*, by default, stores the relevant *ISPyB* link on the crystal page, but also allows the notification of other storage locations on its crystal page. While in theory the data could be uploaded directly into the LIMS and attached to the protein of interest, common practice is either to leave it in place at the synchrotron or to archive it on other IT infrastructures, with both options being more suited than the LIMS for the long-term archival of large quantities of data. Either way, the location of these raw data needs to be recorded in the LIMS to allow retrieval and access later.

## 3. Concluding remarks and future perspectives

The mature nature of routine macromolecular crystallography studies, and the large quantity of data generated over many years, make it suitable to use a LIMS for data management, as opposed to an ELN or manual recording. The LIMS provides significant benefits to researchers by organizing and linking all essential data throughout the life cycle of the project. A complete chain of custody from protein sequence through to structure deposition (Fig. 1 ▸) ensures that the information of all experiments is captured and correctly linked together. Some data import can be automated. However, multiple steps of the workflow, such as crystal-handling information, will require user input. The LIMS can also inform the researchers when key information has not yet been provided, as well as enforcing certain data-entry requirements. Such enforcement can be rigid, preventing further progress until all mandatory information at the current step has been provided. Alternatively, as with *IceBear*, users can be alerted to missing information but can provide it later, up until the point when it is needed; for example, a protein must have a ‘protein acronym’ for synchrotron-shipment submission, but the lack of one should not prevent drop viewing, crystal harvesting or even shipment assembly involving that protein.

The LIMS is also an important educational tool, helping young researchers by recording key data that are of critical importance later in the process of crystal structure determination, refinement, deposition and publication (Figs. 2 ▸ and 3 ▸). The LIMS can also help researchers to fulfill their FAIR data obligations by making all information on the completed work available in an open-access form once the project has been completed. There is a growing expectation that published data should adhere to FAIR data principles. At the most basic level, a LIMS can allow the project to be made public at its conclusion, but this is unlikely to meet the FAIR criteria. In the case of *IceBear*, future work will make adherence to these principles significantly easier than at present.

Both an ELN and a LIMS have the same IT-related risks, namely security breaches, data loss and data corruption. Many of these risks can be mitigated by taking advantage of managed application hosting, where available. The University of Oulu offers managed *IceBear* hosting through its *IceBox* service, handling both the security of the server and the routine backup of scientific data, as well as updates to *IceBear* itself. This hosted service, which includes an automatic backup utility, allows a crystallization facility to optimally benefit from the *IceBear* technology, without any management or installation requirements for *IceBear* itself. If automatic imaging systems are to be incorporated, a script can be provided to push images and plate metadata to the hosted *IceBear* instance; this can run on the imager computer itself or on a dedicated low-power computer such as a Raspberry Pi. A standalone in-house installation is free of charge and is facilitated by a dedicated installer. The standalone instance setup requires a computer running Ubuntu Server and some general expertise. Each *IceBear* installation includes context-sensitive help pages (Fig. 4 ▸), and a demo site is available for testing the system before deciding whether to set it up for the facility (https://www.icebear.fi).

A further risk with any IT-based system is that the product becomes unsupported, leaving researchers’ data locked in a proprietary legacy system with no realistic prospect of migration to a replacement. This scenario has been specifically considered from the beginning of *IceBear* development, both in licensing and in the choice of supporting technologies. *IceBear* is made available as open-source software under the MIT License, which specifically grants the right to modify and redistribute the software. It runs on the industry-standard ‘LAMP stack’ (Linux, Apache, MySQL, PHP) and uses the minimum amount of third-party code, aiming to maximize future maintainability by minimizing the specialist knowledge required. The ongoing work to improve the adherence of *IceBear* to FAIR data principles will make it easier to migrate the data stored therein to any future replacement system.

The metadata-exchange protocols between the home laboratory and synchrotron will make it possible to extend the metadata that are sent from the home-laboratory LIMS to the synchrotron *ISPyB* with sequence, structure and ligand information. This information is required by the downstream structure-solution and structure-analysis pipelines in place at several synchrotrons and therefore these extensions will allow the best use of the impressive quantity of metadata that is generated by the synchrotrons.

In summary, the use of the LIMS will help researchers to keep track of their experiments (Fig. 4 ▸), while also providing optimal benefit from the available experimental facilities (and their computational resources) and allowing adherence of the information that is generated to the FAIR principles.

## Figures and Tables

**Table 1:** Examples of LIMS systems used for crystallography

LIMS	Key features	URL	References
*CRIMS*	Developed at EMBL, providing, as a remote service, automated communication between crystallization-setup and synchrotron data-collection facilities, enabling uninterrupted information flow over the whole sample cycle from pure protein to diffraction data	https://www.embl.org/services-facilities/grenoble/high-throughput-crystallisation/	Cornaciu *et al.* (2021 ▸)
*IceBear*	Used in home laboratories and crystallization facilities to monitor crystallization results and to record all information from crystallization via data collection at synchrotrons to structure deposition and publication	https://icebear.fi/	Daniel *et al.* (2021 ▸)
*ISPyB*	Used at European synchrotrons to collect information from users concerning their crystals and to provide the results of the data collection and data processing by the beamline to users	https://ispyb.esrf.fr/ispyb/overviewPage.do	De Maria Antolinos *et al.* (2015 ▸); Delagenière *et al.* (2011 ▸); Fisher *et al.* (2015 ▸)
*Rock Maker*	Software to monitor the crystallization experiments performed by Formulatrix crystallization-drop imaging systems	https://formulatrix.com/protein-crystallization-systems/rock-maker-crystallization-software/	—
*SG-LIMS*	LIMS to manage data at Argonne National Laboratory and capture data from cloning to crystallization	https://www.anl.gov/event/sglims-a-laboratory-information-management-system-for-macromolecular-crystallography	—
